# High-resolution Hi-C maps highlight multiscale 3D epigenome reprogramming during pancreatic cancer metastasis

**DOI:** 10.1186/s13045-021-01131-0

**Published:** 2021-08-04

**Authors:** Bo Ren, Jinshou Yang, Chengcheng Wang, Gang Yang, Huanyu Wang, Yuan Chen, Ruiyuan Xu, Xuning Fan, Lei You, Taiping Zhang, Yupei Zhao

**Affiliations:** 1 Department of General Surgery, State Key Laboratory of Complex Severe and Rare Diseases, Peking Union Medical College Hospital, Chinese Academy of Medical Sciences, Peking Union Medical College, Beijing, 100023 People’s Republic of China; 2 Annoroad Gene Technology (Beijing) Co., Ltd, Beijing, 100176 People’s Republic of China

**Keywords:** Hi-C, Pancreatic cancer, Metastasis, Epigenetics, Multi-omics

## Abstract

**Background:**

Pancreatic cancer’s poor prognosis is caused by distal metastasis, which is associated with epigenetic changes. However, the role of the 3D epigenome in pancreatic cancer biology, especially its metastasis, remains unclear.

**Methods:**

Here, we developed high-resolution 3D epigenomic maps of cells derived from normal pancreatic epithelium, primary and metastatic pancreatic cancer by in situ Hi-C, ChIP-seq, ATAC-seq, and RNA-seq to identify key genes involved in pancreatic cancer metastasis

**Results:**

We found that A/B compartments, contact domains, and chromatin loops changed significantly in metastatic pancreatic cancer cells, which are associated with epigenetic state alterations. Moreover, we found that upregulated genes, which were located in switched compartments, changed contact domains, and metastasis-specific enhancer-promoter loops, were related to cancer metastasis and poor prognosis of patients with pancreatic cancer. We also found that transcription factors in specific enhancer-promoter loop formation were also associated with metastasis. Finally we demonstrated that LIPC, looped to metastasis-specific enhancers, could promote pancreatic cancer metastasis.

**Conclusions:**

These results highlight the multiscale 3D epigenome reprogramming during pancreatic cancer metastasis and expand our knowledge of mechanisms of gene regulation during pancreatic cancer metastasis.

**Supplementary Information:**

The online version contains supplementary material available at 10.1186/s13045-021-01131-0.

## Background

The incidence of pancreatic cancer has steadily risen in recent years. Despite the significant progress in the treatment of most human cancers, pancreatic cancer continues to be a deadly malignancy due to its distant metastasis and difficulty in early diagnosis. Currently, the 5-year survival rate of patients with distant metastasis is only 3% [1], which is much smaller than that of patients with localized lesions. Therefore, the identification of fundamental mechanisms involved in pancreatic cancer metastasis would provide valuable information for its diagnosis and treatment.

It is well known that genetic mutations play pivotal roles in primary tumorigenesis; however, no “metastasis-specific” genetic mutations have been identified [2]. A recent whole-genome sequencing study showed that there are few heterogeneities of known driver mutations in primary and metastatic pancreatic cancer tissues [3], indicating that genetic mutations per se may have relatively less influence on metastasis. Increasing knowledge of epigenetics has shown that DNA methylation and histone modification are associated with pancreatic cancer pathobiology and subtyping [4], and they change significantly during pancreatic cancer progression. However, as an integral part of epigenetic information [5], the 3D organization of chromatin and its reprogramming during tumor metastasis remain to be elucidated, and even less is known about pancreatic cancer.

Appropriate gene expression is determined by correct chromatin folding. Hi-C, a genome-wide chromosome conformation capture assay, showed that the human genome is folded three-dimensionally in the nucleus and divided into several active or inactive compartments, named A or B [6]. Subsequent analyses showed that compartments are partitioned into ~ 1 Mb in size, called “topologically associating domains” [7]. With increased sequencing depth, high resolution Hi-C data showed that there are contact domains located in megabase-sized chromatin domains [8] and allowed the detection of loops across the entire genome. These chromatin loops are usually mediated by CCCTC-binding factor (CTCF) [8], and often connect regulatory regions, such as enhancer-promoter loops. Previous studies demonstrated that aberrant chromatin interactions contribute to tumorigenesis [9, 10], but no detailed studies have delineated a 3D epigenomic map of cancer metastasis, especially for pancreatic cancer.

To investigate the 3D epigenomic features of pancreatic cancer metastasis, we performed multi-omic analyses of normal pancreatic and pancreatic cancer cells derived from the primary site and liver metastasis. We first analyzed chromatin interactions by high-resolution in situ Hi-C, which is useful for mapping compartments [6], contact domains, and chromatin loops [8]. Moreover, the epigenetic states of chromatin interacting regions were characterized by chromatin immunoprecipitation sequencing (ChIP-seq) of several histone marks and CTCF to characterize contact domains and loops mediated by CTCF or cis-regulatory elements (e.g., enhancer, insulator) by combining the ChIP-seq and Hi-C data. In addition, we performed Assay for Transposase-Accessible Chromatin using sequencing (ATAC-seq) to identify nucleosome-depleted regions (NDRs) and infer essential transcription factors involved in chromatin loops. Finally, transcription profiles were determined by RNA-seq. These sequencing data and integrated analyses enabled to (a) develop comprehensive 3D epigenomic maps, (b) investigate the epigenetic state of the compartments and contact domains, and (c) identify and characterize metastasis-specific enhancer-promoter loops. This characterization of the 3D epigenomic features provided more detailed epigenetic mechanisms of pancreatic cancer metastasis.

## Methods

### Cell culture and transfection

Normal human pancreatic epithelial cells (HPNE, ATCC®CRL-4023), primary pancreatic cancer cells (PANC-1, ATCC®CRL-1469), and metastatic pancreatic cancer cells (Capan-1, ATCC®HTB-79) were obtained from American Type Culture Collection (ATCC) (https://www.atcc.org/). All cell lines were cultured under recommended conditions and were authenticated by high-resolution small tandem repeats (STR) profiling. The siRNA targeting LIPC and a scramble control siRNA were purchased from Guangzhou RiboBio Co., LTD. (Guangzhou, China). Sequences of siLIPC is 5′-GCAAAGGAATTGCTAGTAA-3′. The full length LIPC cDNA was subcloned into the GV658 empty vector (https://www.genechem.com.cn/index/supports/zaiti_info.html?id=208). Transient transfections were performed by lipofectamine 3000 (Invitrogen) according to the corresponding protocol.

### Lentivirus production and infection

Lentivirus for LIPC stably overexpression and shRNA targeting LIPC were purchased from GENECHEM as viral particles with firefly luciferase cassette and puromycin resistance gene. Infection of PANC-1 and Capan-1 was performed according to the manufacturer's protocol. After infection, cells were selected by 2 µg/mL puromycin and maintained by 1 µg/mL puromycin.

### qRT-PCR

Total RNA was extracted by RNA-Quick Purification Kit (ES Science, RN001) and reverse transcription was performed using the Takara PrimeScript™ reagent Kit with gDNA eraser (RR047A). The PCR primers for LIPC are F: ATCAAGTGCCCTTGGACAAAG, R: TGACAGCCCTGATTGGTTTCT. GAPDH primers as internal control were purchased from Sangon Biotech.

### Western blot analysis

Western blot analysis was performed as described previously [11]. Briefly, we performed SDS-PAGE to separate protein lysates (20 µg). Then we used primary and secondary antibodies to detect target proteins, and used an enhanced chemiluminescence assay to visualize protein bands. LIPC antibody was purchased from ProteinTech (21133-1-AP, ProteinTech). β-actin and GAPDH antibodies were purchased from LabLead (A0101-1, G0100, LabLead). E-cadherin, N-cadherin, Vimentin antibodies were purchased from Cell Signaling Technology (3195 T, 13116 T, 3390S, CST).

### Wound-healing assay

The gaps between cells at 0 h were created by Culture-Insert 2 Well (80241, ibidi). The cells were then incubated in medium with 1% FBS, and images were captured at 0 h, 24 h, 36 h, and 48 h. The relative area of cell migration from the gap at 0 h was determined as the migration rate.

### Transwell assay

Transwell assays were conducted as described previously [12]. In brief, 3.0 × 10^4^ PANC-1 or 2.0 × 10^5^ Capan-1 in FBS-free medium were placed into the upper chamber coated with FBS-free medium for migration, or 1:40 diluted matrigel for invasion, and completed medium were placed into the lower chamber. After 24 h, the migrated or invaded cells were fixed by 4% paraformaldehyde and stained with crystal violet. After the membranes were dried, the cell numbers were calculated in 5 high power fields per transwell unit. The mean values were determined in triplicate assays.

### Clinical specimens and immunohistochemistry (IHC) analysis

Eighty-seven primary pancreatic cancer tissues with 81 paired adjacent normal pancreatic tissues were collected from pancreatic cancer patients who undergone radical resection of pancreatic cancer. 27 liver metastasis of pancreatic cancer tissues were collected from pancreatic cancers who undergone liver nodule biopsy. All patients did not receive neoadjuvant therapy. Clinicopathological features of these patients were listed in Additional file 7: Tables S1–S2. This study was approved by the Medical Ethical Committee of Peking Union Medical College Hospital.

Antibody against LIPC (21133-1-AP, ProteinTech) was used to perform IHC analysis. Two experienced pathologists independently assessed the results. Staining intensities were graded as 0 (negative), 1 (low), 2 (medium), or 3 (high), whereas the staining extent was scored from 0 to 100%. The final IHC score = intensity score × percentage score × 100.

### Hi-C library construction and data analysis

In situ Hi-C experiments of 3 cell lines were performed following the protocol provided by Rao et al. [8] with minor modifications. Briefly, the cells were crosslinked by 1% formaldehyde and then resuspended in lysis buffer (10 mM Tris–HCl pH 8.0, 10 mM NaCl, 0.2% Igepal CA-630, 1/10 vol. of proteinase inhibitor cocktail (Sigma)). The genome was then digested by the 4-cutter restriction enzyme *MboI*, and the DNA ends were marked by biotin-14-dCTP and ligated by the ligation enzyme. Next, the nuclear complexes were treated with proteinase K at 65 °C for reverse crosslinking, and the DNA was purified by phenol–chloroform extraction and sheared into 200–600 bp fragments by sonication, and the biotin-labeled DNA fragments were enriched by streptavidin C1 magnetic beads. The Hi-C library from the beads was sequenced on the Illumina HiSeq X Ten platform with 150 bp paired-end reads, and the reads were trimmed to 50 bp.

**Fig. 1:**
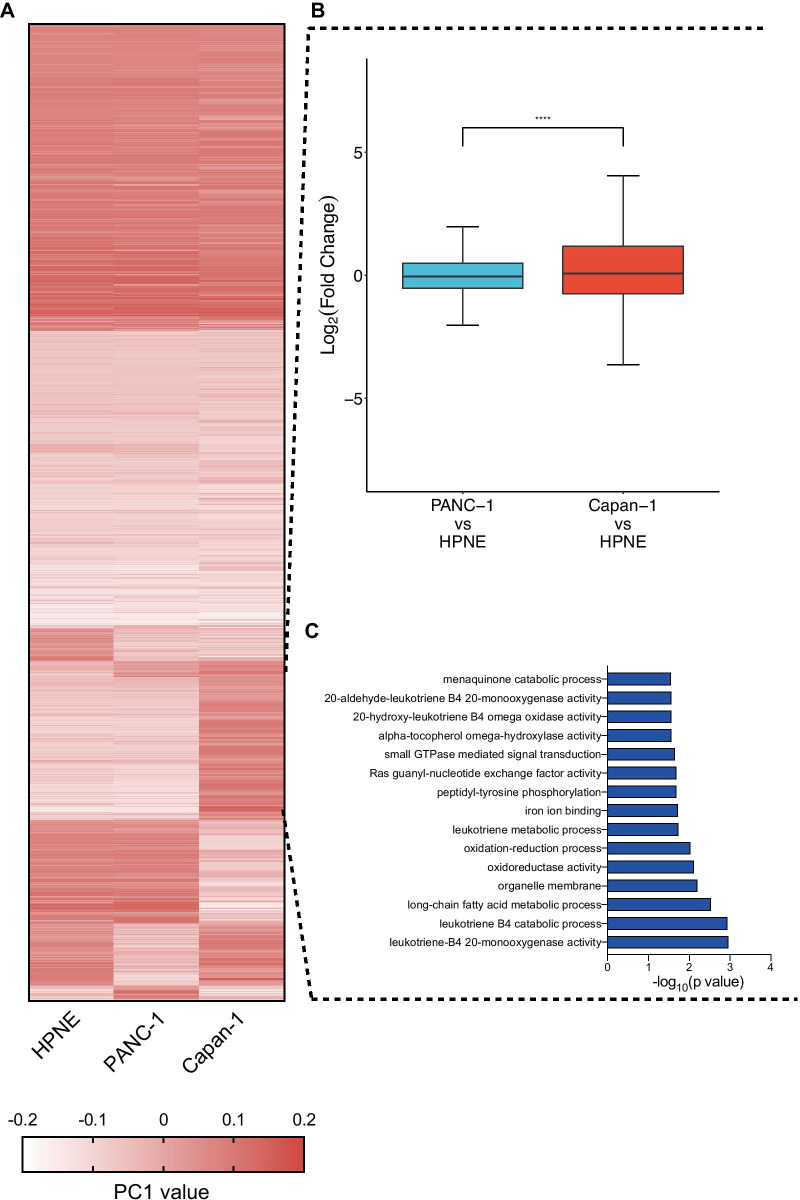
Compartment reorganization during pancreatic cancer metastasis. **a** Cluster analysis of compartments according to PC1 values. **b** Left: Log_2_ (fold change) between primary cancer (PANC-1) and normal (HPNE) cells. Right: Log_2_ (fold change) between metastatic cancer (Capan-1) and normal (HPNE) cells (Wilcoxon rank-sum test, ****p < 0.0001). **c** GO enrichment for concordance genes located in B compartments of primary and normal cells but in A compartments of metastatic cancer cells. Whiskers of box plots are from Q1-1.5*IQR to Q3 + 1.5*IQR

Raw Hi-C fastq files were processed by HiC-Pro [13] for filtering, mapping and creating raw contact count matrices. The matrices were normalized by using the iterative correction method. The stratum-adjusted correlation coefficients (SCC) among libraries were calculated by the HiCRep package [14] and shown in Fig. 1 to confirm that Hi-C libraries were of high quality and reproducible. The replicate datasets of each cell line were pooled to increase the sequence coverage.

### 3C-qPCR

3C-qPCR assays were performed in PANC-1 and Capan-1 cells according to protocol provided by Hagege et al. [15]. After crosslinking of cells, we used the restriction enzyme HindIII to digest genomic DNA, and designed the primers within 50 kb upstream of HindIII restriction site. The 3C ligation products were quantified by SYBR Green-based PCR. Negative control primers were internal primers of GAPDH, which was used for normalization. The primer sequences are listed in the Additional file 7: Table S4.

#### ChIP-seq and ChIP-qPCR

ChIP experiments were performed by following the protocol of the SimpleChIP®Plus Enzymatic Chromatin IP Kit (Cell Signaling Technology #9005). Ten micrograms of chromatin of each of the 3 cell lines was precipitated by the corresponding 10 µL of antibody (CTCF Cell Signaling Technology #3418; H3K4me3 Cell Signaling Technology #9751S; H3K27ac Cell Signaling Technology #8173S; H3K36me3 Cell Signaling Technology #4909S; H3K9me3 Cell Signaling Technology #13969S; H3K27me3 Cell Signaling Technology #9733S) with the recommended dilution rate provided by Cell Signaling Technology. ChIP-seq libraries were sequenced on an Illumina HiSeq X Ten sequencer with paired-end 50-bp reads. All ChIP-seq reads were aligned to human hg19 genome by Bowtie ver 2.1.0 (http://bowtie-bio.sourceforge.net/bowtie2/index.shtml). Peak calling was performed by MACS2 ver 2.1.1 (https://github.com/taoliu/MACS/), followed by peak annotation by using bedtools ver 2.20.1 (http://bedtools.readthedocs.io/en/latest). The Pearson correlation between libraries was performed by plotCorrelation modules from deepTools ver 2.5.3 (https://github.com/deeptools/deepTools).

ChIP-qPCR was performed by SYBR Green dye after ChIP experiments. Primers spanning every enhancer and promoter region were designed and used for ChIP-qPCR. The primer sequences are listed in the Additional file 7: Table S3.

#### ATAC-seq

For each cell line, approximately 50,000 living cells were taken for library construction. The cells were lysed in 1 × lysis buffer to obtain the nuclei, and the transposase-treated libraries were constructed using the TruePrepTM DNA Library Prep Kit V2 for Illumina (Vazyme Biotech, China). PCR was performed to amplify the libraries.

The libraries were sequenced on the Illumina HiSeq X Ten sequencer in paired-end 150 bp style. After removal of adaptor-polluted or low-quality reads, clean data were mapped to the human h19 genome by Bowtie2 ver 2.1.0 (http://bowtie-bio.sourceforge.net/bowtie2/index.shtml). Peaks corresponding to the open region were called by MACS2 using the default parameters. Pearson correlation between libraries was performed by plotCorrelation modules from deeptools.

#### RNA-seq

RNA-seq was performed in triplicate for HPNE, PANC-1, and Capan-1 cells. RNA was extracted using TRIzol reagent (Cat # 15596-018; Thermo Fisher Scientific, NY, USA), and the quality of RNA was assessed by the Bioanalyzer 2100 (Agilent Technologies, CA, USA). A total of 2 µg of RNA per sample was used as input material for sample preparation. Sequencing libraries were constructed by NEBNext_®_ Ultra™ RNA Library Prep Kit for Illumina_®_ (#E7530L, NEB, USA) and sequenced on the Illumina HiSeq X Ten platform with 150 bp paired-end reads. Reads were mapped to the human hg19 genome using HISAT2 ver 2.1.0 (https://github.com/DaehwanKimLab/hisat2). The expression levels of genes in each sample were quantified by HTSeq ver 0.6.1 (https://github.com/simon-anders/htseq) and normalized by the FPKM method. Differentially expressed genes were identified by DESeq2. The p-value was calculated by the Wald test and corrected by Benjamini and Hochberg’s method as the *q*-value. Genes with adj. *p* ≤ 0.05 and |log_2_(fold change)|≥ 1 were identified as differentially expressed genes. Wilcoxon rank-sum tests were performed to compare the expression levels of genes in histone-mark-enriched TADs and looped to enhancers. The annotation was performed by stringtie ver 1.3.3b (https://github.com/gpertea/stringtie). Spearman correlation analysis was performed on the Fragments Per Kilobase of transcript per Million mapped reads (FPKM) values for Additional file 7: Fig. S1C.

#### Identification of A/B compartments

We performed principal component analysis (PCA) on correlation Hi-C heatmaps with 1 Mb resolution to obtain PC1, PC2, and PC3 values by cword-dekker software (https://github.com/dekkerlab/cworld-dekker) with matrix2compartment.pl. The PC1 values combined with gene density and the plaid pattern in the correlation heatmaps along each chromosome to obtain the final ‘PC1’ list. The positive values were set to ‘A’, and negative values were set to ‘B’ based on their association with gene density. The profiling of ChIP-seq and ATAC-seq for compartments was performed by deepTools.

#### Identification of contact domains and contact domain boundaries

We used the Arrowhead [8] module from Juicer software to identify contact domains and contact domain boundaries at 5 kb Hi-C matrices. The profiling of ChIP-seq for contact domain boundaries was performed by deepTools, and we extracted genes located in the ± 5 kb region of the contact domain boundaries for GO enrichment analysis. Common contact domains were defined as domains that had over 80% overlap between cell types. Histone mark-enriched contact domains were defined as contact domains intersecting with corresponding ChIP-seq peaks having the top 25% ChIP-seq signals. Intersections were performed using the Bedtools and BamCompare functions from deepTools. Wilcoxon rank-sum tests were performed to compare the size of contact domains and expression (FPKM) of genes located in corresponding contact domains.

#### Characterization of loops

Using the 5 kb resolution normalized contact count matrices, intrachromosomal loops of each cell line were detected using the HICCUPS [8] program. The regulatory elements were defined as below to find enhancer-promoter loops: (1) Promoters: − 2 kb windows of the TSSs of all expressed genes overlapping with H3K4me3 peaks; (2) Enhancers: H3K27ac peaks located greater than 2 kb from the TSS of all genes; (3) Insulators: CTCF peaks without overlap with the sets of promoters or enhancers. CTCF-mediated loops were defined as loops whose anchors overlapped with CTCF ChIP-seq peaks. For Figs. 4 and 6, we first identified cell type-specific or common enhancers, and then we used our Hi-C loop data to find genes looped to these enhancers. Then, we intersected the H3K27ac ChIP-seq peaks in these enhancers or H3K4me3 ChIP-seq peaks in the promoters of these genes with ATAC-seq peaks to identify enhancer NDRs or promoter NDRs, respectively, for further motif analysis.

#### Motif analysis

We used MEME (http://meme-suite.org/tools/meme) to discover known motifs at enhancer and promoter NDRs under enhancer-promoter loop anchors in 3 cell lines. The top 15 enriched motifs in these NDRs were identified by *p* values.

#### CRISPR interference mediated enhancer repression

short guide RNAs (sgRNAs) used in CRISPR interference (CRISPRi) were designed by CRISPOR [16]. The sgRNAs targeting LIPC candidate enhancers and promoter (listed in Additional file 7: Table S5) were cloned into pX330a dCas9-KRAB vector (addgene # 92361) [17]. Capan-1 cells were transfected by the recombinant vectors with Lipofectamine 3000 according to the manufacturer's protocol (Thermo Fisher Scientific). After 48 h transfection, cells were harvested using RNA-Quick Purification Kit (ES Science, RN001) and LIPC expression was detected by qRT-PCR.

#### Orthotopic xenograft tumor mouse models

NOD-Prkdc^scid^Il2rgtm1/Vst (NPG) mice (female, aged 5–6 weeks) were purchased from Beijing Vitalstar Biotechnology for orthotopic xenograft model construction. After lentivirus infection, 5 × 10^6^ pancreatic cancer cells were inoculated in situ into mice and monitored by an IVIS imaging system. After 6 weeks, mice were sacrificed and liver samples were obtained to evaluate liver metastasis by the IVIS imaging system. The whole process of dissection and ex vivo imaging of liver was completed in 7 min for each mouse. The excised tissues were fixed by 4% poly-formaldehyde overnight and then performed HE staining.

#### Data mining in the cancer genome atlas and gene expression omnibus

A total of 181 pancreatic samples with RNA-seq data and survival data were extracted from the National Cancer Institute GDC Data Portal (https://portal.gdc.cancer.gov/). Four tumor-adjacent normal samples and 1 metastatic cancer sample were excluded from the survival analysis. To determine the association of the expression of key gene sets (genes located in common contact domains switched from inactive types to active types for Fig. 3c, and the genes looped to metastasis-specific enhancers for Fig. 4d) with patient survival, we first performed GSVA [18] and stratified patients into low enrichment (GSVA score ≤ median) and high enrichment (GSVA score > median) groups. Then, we performed Kaplan–Meier survival analysis, and the log-rank test p values were used to determine significance.

To identify differential expressed genes in pancreatic cancer metastasis, gene expression data from GSE42952 [19] (microarray), GSE63124 [20] (RNA-seq), and GSE71729 [21] (microarray) were downloaded. After removing outlier samples (defined by PCA), differentially expressed genes were identified by DESeq2 (GSE63124) and limma (GSE42592, GSE71729) R package, and defined as genes with adj. *p* ≤ 0.05 and |log_2_(fold change)|≥ 1.

## Results

### Global reprogramming of the 3D epigenome during pancreatic cancer metastasis

To investigate the 3D organization of chromatin of pancreatic cancer, we created high-resolution Hi-C maps of normal pancreatic cells (HPNE) and pancreatic cancer cells derived from the head of pancreas (PANC-1) and liver metastasis (Capan-1) by in situ Hi-C. After sequencing filtering, we generated over 3 billion valid paired-end reads from Hi-C data of three cell lines (Additional file 1). After confirming the reproducibility of biological replicates (Additional file 7: Fig. S1A), we merged replicates to maximize Hi-C matrix resolution for each cell line. There were much more than 1000 Hi-C contacts in 80% of bins of 5 kb Hi-C matrices (Additional file 7: Fig. S2), enabling examination of chromatin interactions at sub-5 kb resolution [8].

To determine other epigenetic features during pancreatic cancer metastasis, we also performed ATAC-seq to evaluate chromosome accessibility and ChIP-seq to identify regulatory elements, including active enhancers (H3K27ac), active promoters (H3K4me3), heterochromatin (H3K9me3), repressive regions (H3K27me3) and transcription elongation regions (H3K36me3), and CTCF-binding sites. To assess the relationship between 3D organization of chromatin and the transcriptome, we performed RNA-seq for 3 cell lines. Similar to Hi-C, the above sequencing data also had high reproducibility among biological replicates (Additional file 7: Fig. S1B–J).

To explore the differences in multi-omics data among 3 cell lines, we first generated correlation heatmaps of sequencing libraries from Hi-C, ChIP-seq, ATAC-seq, and RNA-seq to investigate changes of 3D epigenomic features among these pancreatic cells. We found that chromatin interactions (Additional file 7: Fig. S1A), chromatin states (Additional file 7: Fig. S1E–J), CTCF-binding sites (Additional file 7: Fig. S1D), chromosome accessibility (Additional file 7: Fig. S1B), and transcriptomic features (Additional file 7: Fig. S1C) of normal HPNE cells were similar to those of primary PANC-1 cells, but metastatic Capan-1 cells showed dramatic changes in the chromatin interactions, chromatin states, chromosome accessibility and transcriptome. These results demonstrated concordance between different types of sequencing and provided preliminary evidence that 3D epigenome reprogramming did exist in pancreatic cancer metastasis; furthermore, the similarity between normal and primary and apparent differences of the 3D epigenome in metastasis were consistent with other epigenetic studies of pancreatic cancer metastasis [20, 22].

### The association between compartment rearrangement and pancreatic cancer metastasis

To investigate chromatin interaction reprogramming in detail and its association with the transcriptome during pancreatic cancer metastasis, we first identified A/B compartments and their changes during pancreatic cancer metastasis by using principal component analysis (eigenvector decomposition) of the Pearson correlation matrix [6]. We found that the compartment switching between metastasis and normal/primary pancreatic cancer cells was more substantial (Fig. 1a, Additional file 2) [26.70% in Capan-1 vs. HPNE (Additional file 7: Fig. S3B) and 30.75% in Capan-1 vs. PANC-1 (Additional file 7: Fig. S3C)], than the compartment switching between normal pancreatic ductal and primary cancer cells (16.17%) (Additional file 7: Fig. S3A).

To investigate the association between these epigenetic features and partitioning of A and B compartments, we tested the relative levels of histone modifications and chromosome accessibility across the compartments. We found that A compartments have higher active histone modifications, including H3K4me3, H3K27ac, and H3K36me3 (Additional file 7: Fig. S3D–F), and chromosome accessibility than B compartments (Additional file 7: Fig. S3G–I), which is similar to the chromatin compartmentalization in mammals observed previously [6]. For the B compartments, all 3 cell lines had higher levels of inactive histone modification. However, H3K9me3 was significantly enriched in the B compartments of HPNE and PANC-1 cells (Additional file 7: Fig. S3D–E), while the B compartments of Capan-1 cells had higher levels of H3K27me3 (Additional file 7: Fig. S3F).

Compartment status is known to be associated with gene expression. Genes in the A compartments tend to be expressed at higher levels than those located in the B compartments (Additional file 7: Fig. S3J–L), which is consistent with a previous report [23]. In addition, the expression of genes located in compartments switching from A to B tended to decrease, while the expression of genes located in compartments switching from B to A tended to increase (Additional file 7: Fig. S3J–L). To investigate the association between compartment rearrangement and pancreatic cancer metastasis, we performed cluster analysis of the PC1 value to identify B compartments of normal and primary cancer cells that changed into A compartments of metastatic cancer cells (Fig. 1a), and genes located in these compartments exhibited higher expression (Fig. 1b). Then, we found genes that were located in these compartments and showed concordance between gene expression and compartment switching (Additional file 2). Gene Ontology (GO) enrichment analysis (Additional file 2) of these genes found that these genes were enriched for Ras/small GTPase-associated terms, which are essential for pancreatic cancer progression [24, 25], and oxidation–reduction associated terms (Fig. 1c), which were found in pancreatic cancer distant metastasis in a previous study [20]. These results suggested that compartment rearrangement may promote pancreatic cancer metastasis.

### Histone modification changes and contact domain splitting during pancreatic cancer metastasis

**Fig. 2:**
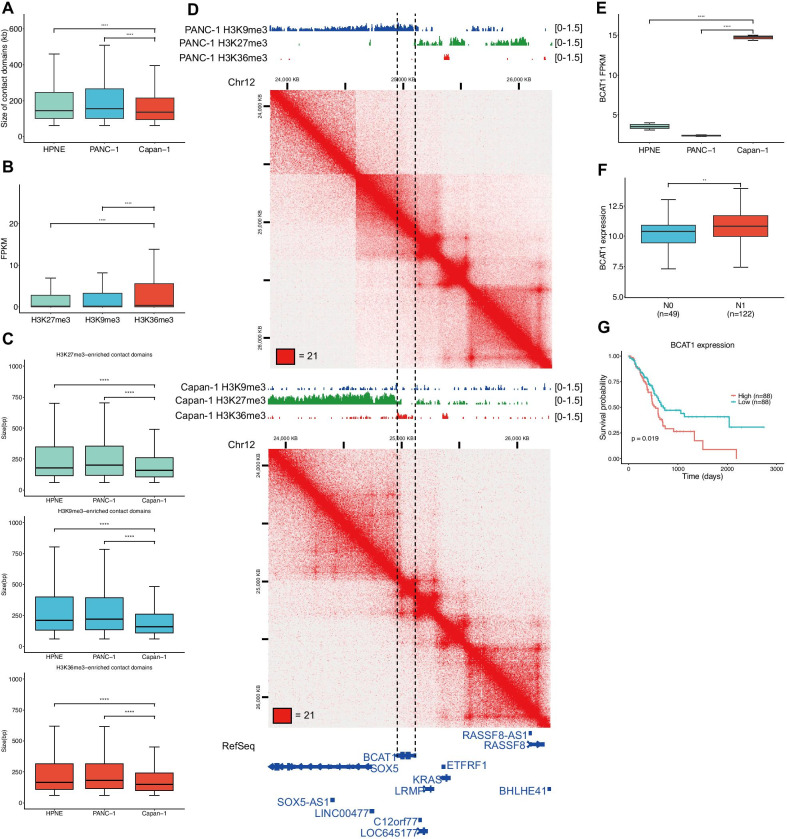
Contact domain reprogramming during pancreatic cancer metastasis. **a** Shown is the size of contact domains in 3 cell lines. **b** Average expression levels of genes (FPKM) located in each histone mark-enriched contact domains and contact domains. **c** Shown is the size of histone mark-enriched contact domains across 3 cell lines (Wilcoxon rank-sum test in a–c, *p < 0.05, ****p < 0.0001). **d** An example of contact domain splitting (between the dashed lines) coupled with chromatin state switching (shown by Hi-C maps and histone ChIP-seq tracks) around BCAT1 locus in Capan-1 and PANC-1 cells (*p < 0.05, **p < 0.01, ***p < 0.001, ****p < 0.0001). **e** BCAT1 expression in normal, primary and metastatic pancreatic cancer cells (****adj. *p* < 0.0001). **f** BCAT1 expression in tumor samples with N0 and N1 classification in TCGA PAAD dataset (Student’s *t* test, **p < 0.01). **g** Kaplan–Meier survival curves for overall survival for BCAT1 expression in the TCGA PAAD dataset. Whiskers of box plots are from Q1-1.5*IQR to Q3 + 1.5*IQR

To investigate contact domain changes during pancreatic cancer metastasis, we used the Arrowhead [8] program to identify contact domains, which is suitable for the high-resolution Hi-C matrices [26]. We identified 6398 contact domains with a mean size of 237 kb in HPNE cells, 6449 contact domains with a mean size of 247 kb in PANC-1 cells, and 8580 contact domains with a mean size of 185 kb in Capan-1 cells (Additional file 3). Interestingly, the size of contact domains in Capan-1 cells became much smaller than that in HPNE (*p* < 0.0001, Wilcoxon rank-sum test) and PANC-1 cells (*p* < 0.0001, Wilcoxon rank-sum test) (Fig. 2a). To explore factors driving the contact domain alterations, we first examined the contact domain boundaries of 3 cell lines (Additional file 3). We found that the numbers of contact domain boundaries of normal and primary cancer cells were nearly identical but increased dramatically during pancreatic cancer metastasis (Additional file 7: Fig. S4A), which was consistent with the decreased size of contact domains in metastatic cancer cells and indicated that new contact domain boundary formation during pancreatic cancer metastasis.

To determine which factors contributed to the formation of new contact domain boundaries, we first calculated the proportion of CTCF-positive contact domain boundaries. We found that the proportion decreased significantly during pancreatic cancer metastasis (Additional file 7: Fig. S3B). Then, we examined the distribution of transcriptional start sites (TSS) around the identified cell-type-specific contact domain boundaries. Several GO terms related to housekeeping genes were strongly enriched around cell type-specific boundaries with high similarity among the 3 cell lines (Additional file 3). Seemingly, genes located at contact domain boundaries per se may not contribute to contact domain splitting during pancreatic cancer. Other factors may mediate the formation of new contact boundaries.

Thus, we then focused on the chromatin state of the contact domains and the association between the chromatin state change and contact domain splitting during pancreatic cancer metastasis. We characterized the contact domains by the integration of ChIP-seq in normal, primary, and metastatic pancreatic cancer cells to demarcate active and inactive regions. We used H3K9me3 to annotate heterochromatic contact domains, H3K27me3 to annotate repressed contact domains, and H3K36me3 to annotate transcriptionally active contact domains (Additional file 3). As expected, the gene expression levels of H3K36me3-enriched contact domains were higher than those of other subgroups of contact domains (Fig. 2b), and the size of H3K36me3-enriched contact domains was the smallest among all subgroups of contact domains (Fig. 2c) (*p* < 0.0001, Wilcoxon rank-sum test). A similar pattern of the size and gene expression levels for the histone-mark-enriched contact domains was identified across all cell types (Additional file 7: Fig. S4C–H) (*p* < 0.0001, Wilcoxon rank-sum test). In addition, similar to the overall trend of smaller contact domains in metastatic cancer cells (Fig. 2a), each subgroup of histone mark-enriched contact domains also decreased during pancreatic cancer (Fig. 2c) (*p* < 0.0001, Wilcoxon rank-sum test). These results showed that contact domains, identified by the Arrowhead program, were enriched with contact domain-associated biological features, which is consistent with a previous report [26], and contact domain splitting can occur in all kinds of epigenetic state-specific contact domains during pancreatic cancer metastasis. Thus, we tested the proportion of each subgroup of histone mark-enriched contact domains in common contact domains and specific contact domains. We found that histone modification patterns are similar in primary and normal common or specific contact domains (Additional file 7: Fig. S3J). However, primary-specific contact domains have a higher percentage of H3K27me3-enriched contact domains compared with metastasis (Additional file 7: Fig. S5I), indicating that inactive contact domains may switch to active contact domains during pancreatic cancer metastasis. For instance, we found that KRAS was located in an H3K36me3-enriched contact domain in both primary and metastatic cancer cells. An H3K9me3-enriched contact domain in primary cancer cells alongside this KRAS contact domain was divided into two contact domains enriched with H3K27me3 and H3K36me3 in metastatic cancer cells. The BCAT1 gene, which is essential for reprogrammed branch-chain amino acid metabolism, was located in the new H3K36me3-enriched contact domain (Fig. 2d), which caused its elevated expression (Fig. 2e). According to The Cancer Genome Atlas (TCGA) datasets, BCAT1 was up-regulated in pancreatic cancer samples with lymph node metastasis compared with those without lymph node metastasis (Fig. 2f) (*p* = 0.02, Student’s *t*-test), and BCAT1 expression was negatively associated with overall survival (Fig. 2g) (*p* = 0.028, log-rank test). Together, these results indicated that contact domain splitting might be coupled with epigenetic state alterations during pancreatic cancer metastasis.

### Chromatin states of common contact domains changed during pancreatic cancer metastasis

**Fig. 3:**
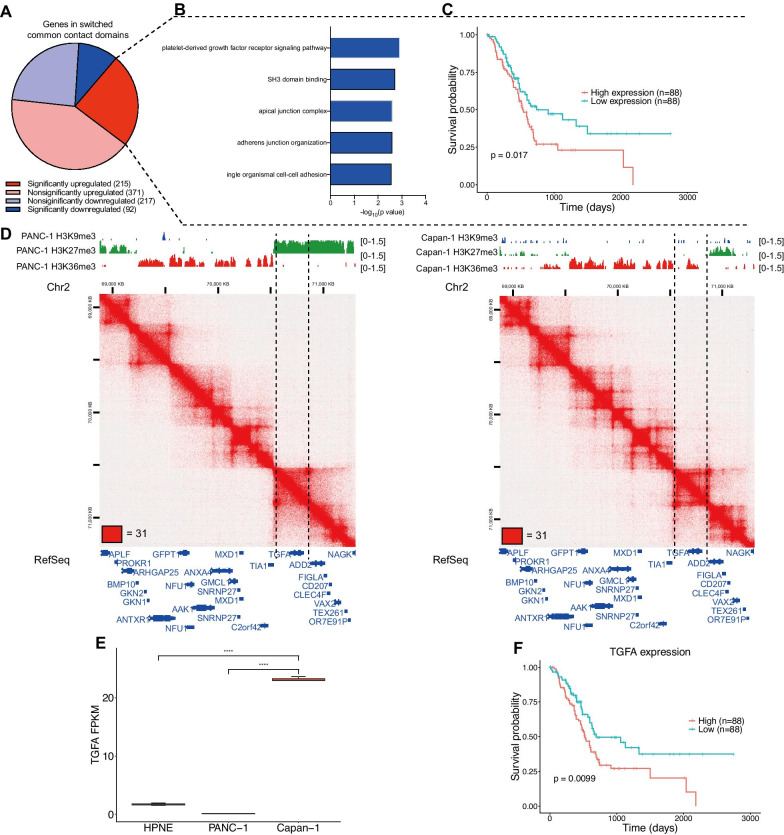
Common contact domains with changed chromatin states during pancreatic cancer metastasis. **a** Pie chart showing that genes located in inactive contact domains of PANC-1 but in active common contact domains of Capan-1 enrich more upregulated genes. **b** GO enrichment of significantly upregulated genes located in common contact domains that switched from inactive type to active type. **c** Kaplan–Meier survival analysis of 176 patients with pancreatic cancer from the TCGA PAAD dataset by calculating the gene set enrichment scores (See material and methods) for the significantly upregulated genes in Fig. 4a. **d** An example of a common contact domain with changed chromatin states (shown by Hi-C maps and histone ChIP-seq tracks) surrounding the TGFA locus (chr2:68,670,001–71,760,001). **e** TGFA expression in normal, primary and metastatic pancreatic cancer cells. **f** Kaplan–Meier survival curves for overall survival for TGFA expression in the TCGA PAAD dataset. Whiskers of box plots are from Q1-1.5*IQR to Q3 + 1.5*IQR

Having demonstrated chromatin state alterations in specific contact domains, we wondered whether these alterations occur in common contact domains during pancreatic cancer metastasis. We focused on the common contact domains between primary and metastatic cancer cells. Common contact domains that enriched H3K9me3 or H3K27me3 were considered inactive common contact domains, and those that enriched H3K36me3 were considered active common contact domains. We extracted the genes located in common contact domains, which were inactive common contact domains in primary cancer and active common contact domains in metastasis. Then, we found that the majority of these genes were upregulated in metastatic cancer cells (Fig. 3a). GO analysis showed that the significantly upregulated genes (log_2_FC ≥ 1 and adj. *p*-value ≤ 0.05) were enriched in the platelet-derived growth factor receptor signaling pathway (Fig. 3b, Additional file 4), which is associated with metastasis. In addition, we used the TCGA cohort of pancreatic cancer patients with gene expression data and long-term follow-up data to determine the correlation between the expression of these genes and patient survival. The analysis showed that high expression of these genes was significantly (*p* = 0.0175, log-rank test) associated with poor survival (Fig. 3c). For example, TGFA, a kind of EGFR ligand associated with pancreatic cancer invasion [27] and poor survival (*p* = 0.0148, log-rank test) (Fig. 3f), was located in an H3K27me3-enriched common contact domain of a primary site but located in an H3K36me3-enriched common contact domain of metastasis with elevated expression (adj. *p* < 0.0001) (Fig. 3d–e). Chromatin state alterations in common contact domains can also promote pancreatic cancer metastasis.

### Genes looped to metastasis-specific enhancers are associated with pancreatic cancer metastasis and poor prognosis

**Fig. 4:**
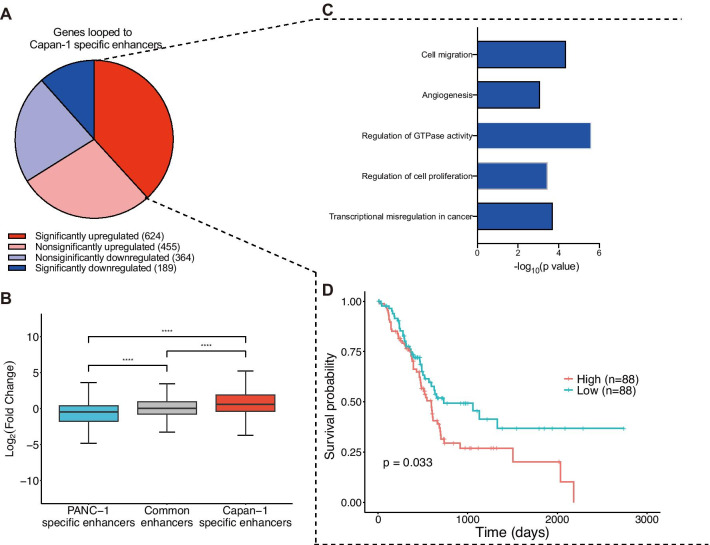
Genes looped to metastasis-specific enhancers are associated with pancreatic cancer metastasis and poor prognosis. **a** Pie chart showing that genes looped to metastasis-specific enhancers enrich more upregulated genes. **b** Log_2_ (fold change) between Capan-1 and PANC-1 cells of genes that looped to primary-specific, common, and metastasis-specific enhancers (Wilcoxon rank-sum test, ****p < 0.0001). **c** Representative GO terms, which are associated with cancer metastasis, of significantly upregulated genes looped to metastasis-specific enhancers. **d** Kaplan–Meier survival analysis of 176 patients with pancreatic cancer from the TCGA PAAD dataset by calculating the gene set enrichment scores for the significantly upregulated genes in a. Whiskers of box plots are from Q1-1.5*IQR to Q3 + 1.5*IQR

To investigate key chromatin contacts involved in pancreatic cancer metastasis, we analyzed our Hi-C data at 5 kb resolution and identified intrachromosomal significant loops in normal, primary, and metastatic cancer cells by using the HiCCUPS program [8]. Similar to the overall trends of contact domains and contact domain boundaries, the number of loops also increased dramatically in metastatic cancer cells (Additional file 7: Fig. S5A, Additional file 5), and the proportion of CTCF-mediated chromatin loops was also significantly decreased in metastatic cancer cells (Additional file 7: Fig. S5B). To characterize regulatory loops, we extracted the loop anchors that overlapped with active promoters, active enhancers, and insulators (definitions of these regulatory elements are listed in the Methods). In the top 20 most frequent chromatin interaction categories, a majority of loop anchors overlapped with these regulatory elements (Additional file 7: Fig. S5C). Cell type enhancers play an essential role in controlling cell-type-specific gene expression programs by engaging in physical contact [28]. Thus, we tested the expression of genes that looped to cell type-specific enhancers. We found that a majority of the genes whose promoters looped to Capan-1-specific enhancers were upregulated compared with primary cancer cells (Fig. 4a). In addition, these genes expressed significantly higher levels than the genes looped to PANC-1-specific enhancers (*p* < 0.0001, Wilcoxon rank-sum test) (Fig. 4b). Similarly, a majority of the genes whose promoters looped to primary-specific enhancers were also upregulated (Additional file 7: Fig. S5D) and were expressed at significantly higher levels than the genes looped to normal-specific enhancers (*p* = 0.00044, Wilcoxon rank-sum test) (Additional file 7: Fig. S5E). These results were consistent with the fact that cell type-specific enhancers contribute to an increase in specific gene expression [28]. We were interested in the association between genes looped to metastasis-specific enhancers and metastasis (Additional file 5). Therefore, we performed GO and KEGG enrichment analyses on significantly upregulated (log_2_FC ≥ 1 and adj. *p* ≤ 0.05) genes that are looped to metastasis-specific enhancers (Additional file 5). Surprisingly, these genes were significantly enriched in cell migration and angiogenesis terms, suggesting that these genes were associated with pancreatic cancer metastasis (Fig. 4c). We also defined these significantly upregulated genes as a gene set to perform a similar gene set variation analysis (GSVA) method to obtain the gene sets enrichment score for each patient with pancreatic cancer from the TCGA cohort (*n* = 176). Kaplan–Meier analysis revealed that high expression of these genes was significantly associated with poor prognosis (*p* = 0.0052, log-rank test) (Fig. 4d). These results demonstrated that changes in loops during pancreatic cancer metastasis could upregulate metastasis-associated genes.

### Hi-C identifies LIPC as a metastasis-promoting gene of pancreatic cancer

**Fig. 5:**
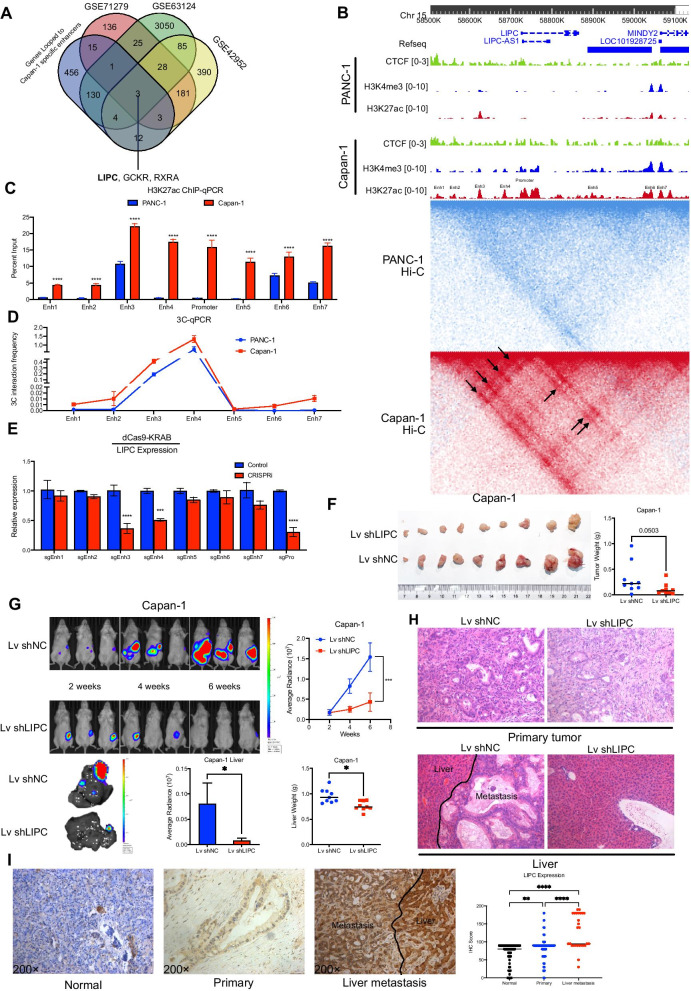
LIPC promotes pancreatic cancer cell migration. **a** Venn diagrams showed that 3 key genes looped to Capan-1 specific enhancers and upregulated in metastatic lesions. **b** In situ Hi-C maps and histone ChIP-seq tracks surrounding the LIPC locus. The enhancer-promoter loops are shown by black arrows. Enh: enhancer. **c** H3K27ac ChIP-qPCR were performed on candidate enhancers shown in b (Two-way ANOVA, ****adj. *p* < 0.0001). **d** 3C-qPCR of the interaction between the candidate enhancers and LIPC promoters. **e** The expression level of LIPC after enhancer silencing by CRISPRi. Enh: enhancer; Pro: Promoter (Multiple unpaired *t* tests, sgEnh3 adj. *p* < 0.0001, sgEnh4 adj. *p* = 0.00012, sgPro adj. *p* < 0.0001). **f** Photographs of dissected tumors from orthotopic xenograft mice injected with Lv shNC and Lv shLIPC Capan-1 cells (Left); Analysis of primary tumor weights were calculated at the end of the experiment (Wilcoxon rank sum test, *p* = 0.0503) (Right). **g** Upper: representative IVIS images of orthotopic xenograft mice and analysis of the average radiance of the mice (Whisker: mean ± SEM, Two-way ANOVA, adj. *p* = 0.0004). Lower: Representative IVIS images of liver samples and analysis of the average radiance of liver samples (Whisker: mean + SD, Wilcoxon rank sum test, *p* = 0.0401, outliers were removed), and the liver weights of the mice (Wilcoxon rank sum test, *p* = 0.0106). (Lower) (H) Representative HE staining pictures of the primary tumor and liver. (Magnification, × 200). **i** Representative images of IHC staining of LIPC in adjacent normal pancreatic tissues, primary pancreatic cancer tissues, and pancreatic cancer with liver metastasis tissues (magnification, × 200). Analysis of LIPC expression according to IHC scores (Kruskal–Wallis test; primary vs. normal, adj. *p* = 0.0019; liver metastasis vs normal, adj. *p* < 0.0001; liver metastasis vs. primary, adj. *p* < 0.0001)

Using high-resolution Hi-C data, we have identified over 600 genes with loop reprogramming and associated with pancreatic cancer metastasis. We next sought to validate our findings in pancreatic cancer tissues. Thus, 3 gene expression datasets from Gene Expression Omnibus (GEO) were downloaded and analyzed to obtain differential expressed genes between tissues of primary pancreatic cancer and distant metastasis. After standardization, we identified significant upregulated genes in distant metastasis from each dataset (392 in GSE71279, 2189 in GSE63124, 706 in GSE42952). After intersecting these genes, we identified 3 key genes that looped to Capan-1 specific enhancers and upregulated in distant metastasis tissue of pancreatic cancer (Fig. 5a). We focused on lipase C (LIPC) gene whose log_2_(fold change) values in all comparison groups are the largest (11.44 in Capan-1 vs. PANC-1, 1.54 in GSE71279, 7.96 in GSE63124, 4.34 in GSE42952). H3K27ac ChIP-seq profiles showed that there are 7 candidate enhancers are located alongside the LIPC locus. These enhancers were looped to LIPC gene (black arrows in the Hi-C heatmap) in Capan-1 but not in PANC-1 (Fig. 5b), which causes LIPC elevated expression in Capan-1. H3K27ac ChIP-qPCR showed that H3K27ac level of all candidate enhancers and LIPC promoters are higher in Capan-1 than those in Capan-1 (Fig. 5c). In addition, 3C-qPCR detected stronger interactions between the LIPC promoter and the enhancers (except for enhancer 5) in Capan-1 (Fig. 5d). These validation results were consistent with sequencing, and enhancer 3 and 4 may have a greater effect on LIPC expression (Fig. 5d). To further confirm the function of the candidate enhancers, we applied the CRISPRi system to silence each candidate enhancer. We found that repression of enhancer 3 and 4 could result in a significant reduction of LIPC expression (Fig. 5e). These results showed that enhancer 3 and 4 exerted important roles on LIPC gene activation.

To test the effect of LIPC on pancreatic cancer metastasis, we used plasmids or lentivirus (Lv) to increase LIPC expression and siRNAs or shRNAs to reduce LIPC expression (Additional file 7: Fig. S6A). Wound-healing assays and transwell assays showed that LIPC overexpression promoted but LIPC knockdown inhibited pancreatic cancer cells migration and invasion (Additional file 7: Figs. S6B–C and S6E). Detection of epithelial-mesenchymal transition (EMT) markers showed that LIPC can promote EMT process of pancreatic cancer cells (Additional file 7: Fig. S6D). Orthotopic xenograft tumor models revealed that the Lv shLIPC group showed significantly reduced tumor burden, compared with Lv shNC group. The primary tumor weights, liver weights, and the pancreatic cancer cells with liver metastasis were also lower in LIPC silenced group (Fig. 5f–h). Similarly, the primary tumor weights and liver weights were higher in LIPC overexpressed group, compared with the control group (Additional file 7: Fig. S6F–H). Furthermore, we performed IHC to detect LIPC expression of clinical specimens containing normal pancreatic tissues, primary/liver metastatic pancreatic cancer tissues. We found that LIPC expression was significantly higher in liver metastasis than primary pancreatic cancer. Similarly, LIPC expressed higher in primary pancreatic cancer than tumor-adjacent normal pancreatic tissues (Fig. 5i). These results were consistent with LIPC expression data from GEO datasets in Fig. 5a. Together, our results demonstrated that loop reprogramming cause LIPC upregulation during pancreatic cancer metastasis, and confirmed an important role of LIPC in pancreatic cancer metastasis clinically and functionally.

### Transcription factors involved in metastasis-specific enhancers looped to promoters

**Fig. 6:**
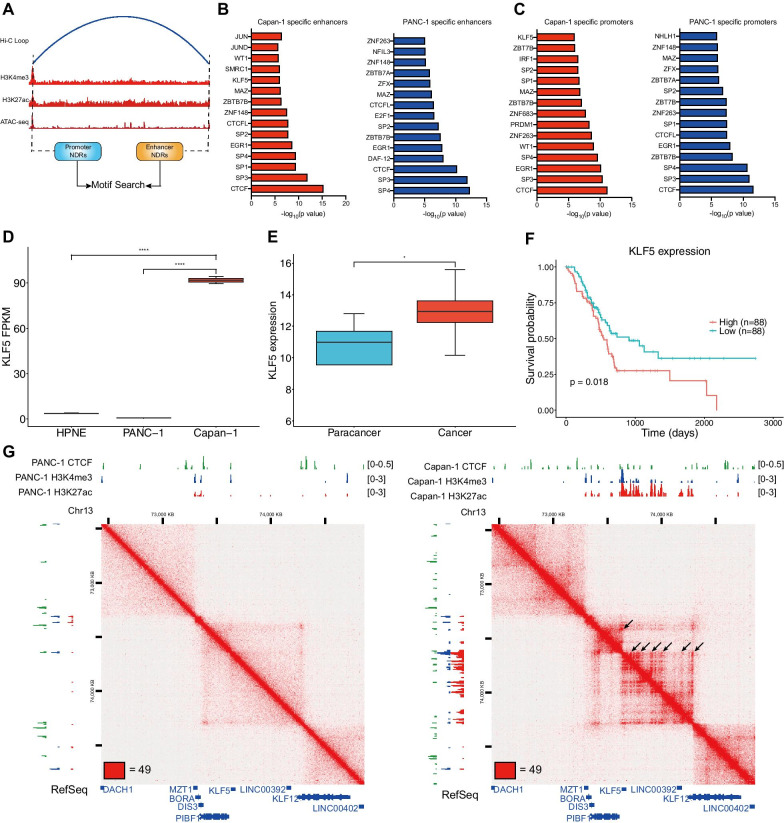
TFs enriched at cell type-specific enhancers that loop to promoters. **a** The workflow of identification of promoter NDRs and enhancer NDRs by integrating ChIP-seq and ATAC-seq data to find TFs. **b** Top most frequent TF-binding motifs found in cell type-specific enhancer NDRs that looped to promoters. **c** Top most frequent TF-binding motifs found in corresponding promoter NDRs. **d** KLF5 expression in normal, primary, and metastatic pancreatic cancer cells (****adj. *p* < 0.0001). **e** KLF5 expression in tumor samples (cancer) and tumor-adjacent normal samples (paracancer) from the TCGA PAAD dataset (Wilcoxon rank-sum test, **p < 0.001). **f** Kaplan–Meier survival curves for overall survival for KLF5 expression in the TCGA PAAD dataset. **g** In situ Hi-C maps and histone ChIP-seq tracks surrounding the KLF5 locus in PANC-1 and Capan-1. The enhancer-promoter loops are shown by black arrows. Whiskers of box plots are from Q1-1.5*IQR to Q3 + 1.5*IQR

Having demonstrated that genes looped to metastasis-specific enhancers were associated with pancreatic cancer metastasis, we wondered which factors contribute to the formation of these enhancer-promoter loops. Transcription factors (TFs) are key regulators that bind to regulatory elements to mediate enhancer-promoter interactions [29]. Thus, we were interested in which TFs mediated loop interactions between promoters and cell-type-specific enhancers. We first identified cell type-specific enhancers that loop to promoters. Next, we integrated our ATAC-seq data to identify NDRs within each cell-type-specific enhancer and corresponding promoter, which can narrow the motif search window to improve the accuracy of TF prediction (Fig. 6a). To explore critical TFs involved in loops between metastasis-specific enhancers and corresponding promoters, we analyzed the subsets of NDRs found in metastasis-specific enhancers involved in enhancer-promoter loops, identifying motifs for TFs such as SP1, EGR1, and KLF5 (Fig. 6b, Additional file 6). In contrast, NDRs in primary pancreatic cancer-specific enhancers involved in enhancer-promoter loops are enriched for motifs for TFs such as E2F1, MAZ, and ZFX (Fig. 6b, Additional file 6). These motif analysis results of NDRs located at corresponding promoters were similar to those located at cell-type-specific enhancers (Fig. 6c, Additional file 6). Notably, all sets of NDRs had CTCF and CTCFL motifs, which was consistent with the fact that CTCF is a vital chromatin structural protein mediating chromatin loops [30], and these enhancers and promoters were selected as the subset of loop anchors identified by Hi-C data.

It is worth noting that the KLF5 motif was enriched at metastasis-specific enhancers and corresponding promoters (Fig. 6b–c). Previous studies demonstrated that KLF5 is critical for pancreatic cancer progression [31, 32]. RNA-seq analysis revealed that KLF5 was significantly upregulated in metastatic cancer cells (adj. *p* < 0.0001) (Fig. 6d). Furthermore, we analyzed the TCGA dataset and found that KLF5 expression was higher in pancreatic cancer samples than that in normal samples (*p* = 0.0017, Student’s *t*-test) (Fig. 6e), and high expression of KLF5 was associated with poor prognosis of patients with pancreatic cancer (*p* = 0.0145, log-rank test) (Fig. 6f). Using our multi-omic data, we identified several enhancers that looped to the KLF5 promoter region in metastatic cancer cells (Fig. 6g, black arrows), which cannot be found in primary cancer cells. Together, metastasis-specific enhancers loop to promoters of several key TFs, which can mediate additional enhancer-promoter loops to upregulate genes associated with pancreatic cancer metastasis.

## Discussion

**Fig. 7:**
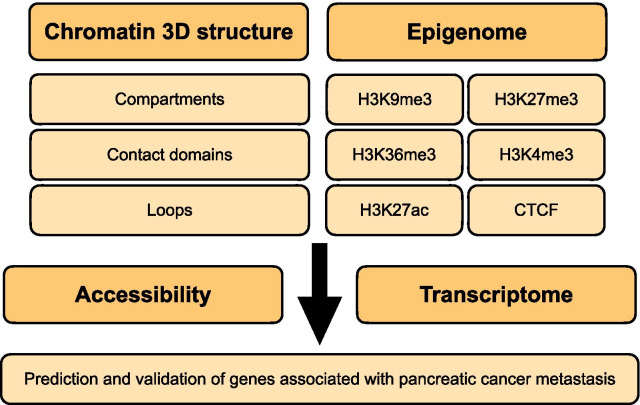
The analytic framework of multi-omics data based on Hi-C. Flow diagram showed the analytic framework in this study to identify 3D epigenome reprogramming associated with key gene transcription during pancreatic cancer metastasis

Previous work had developed 3D chromatin structure of pancreatic cancer, but they focused on structural variants detection [33] by Hi-C and TAD alterations [34, 35]. Here, we reported sub-5-kb resolution Hi-C maps to identify contact domain and loop alterations during pancreatic cancer metastasis. By combining with other sequencing, we provided an analytical framework integrating multi-omics data based on Hi-C to study 3D epigenome and transcriptional regulation (Fig. 7), and demonstrated that the 3D epigenome reprogramming played an important role in pancreatic cancer metastasis. Sub-5-kb Hi-C maps allowed us to identify compartments, contact domains, and loops simultaneously. Together with the other epigenomic and transcriptomic datasets, these high-resolution Hi-C map provided a useful resource for the pancreatic tumor field and for future studies on the relationships between chromatin interaction, epigenetic factors, and transcriptional regulation.

Before analyzing the multi-omics data, we first examined the correlation among these omics data to test the data quality. We found that A compartments exhibited active chromatin state and higher gene expression. Genes located in H3K36me3-enriched contact domains expressed higher than those in other contact domains. Meanwhile, genes looped to cell-type-specific enhancers exhibited higher expression in the corresponding cell type. These results confirmed the reliability of the Hi-C maps.

Our Hi-C maps highlighted that pancreatic cancer cells derived from liver metastasis exhibited dramatic differences in different chromatin scale. We found that compartment rearrangement in metastatic pancreatic cancer cells was more substantial. In addition, contact domains altered genomically and epigenomically during pancreatic cancer metastasis. Our results showed that contact domains became smaller and more numerous, which was associated with changes of histone modifications, during pancreatic cancer. Finally, loops increased dramatically in metastatic pancreatic cancer cells. Meanwhile, we identified over 600 genes looped to metastasis-specific enhancers and TFs involved in these loops. These results showed that 3D epigenome reprogramming did exist during pancreatic cancer metastasis. Notably, by combining histone modifications data, we found that (1) different distribution patterns of H3K9me3 in compartments and contact domains, indicating that reprogramming of large organized chromatin histone H3 lysine 9 (H3K9)-modified heterochromatin (LOCKs) during pancreatic cancer metastasis [20] can be accompanied by reprogramming of compartments and contact domains (Fig. 2d); (2) enhancer reprogramming during pancreatic cancer metastasis [22] can be accompanied by loop reprogramming (Fig. 5b). These findings validated and expanded the knowledge of previous studies.

Furthermore, clustering analysis showed that genes located in regions with changed chromatin interactions were associated pancreatic cancer metastasis. On the one hand, more than 90% pancreatic cancer patients carry KRAS mutation [24, 36], which activates Ras signaling pathway constitutively and promotes pancreatic cancer tumorigenesis and metastasis. GO enrichment analysis showed that genes with compartment switching, contact domain changing, and loop reprogramming were enriched at KRAS-associated terms (e.g., small GTPase mediated signal transduction, SH3 domain binding, regulation of GTPase activity, etc.). On the other hand, these genes were enriched at terms that are associated with adaptation to hepatic microenvironment, due to Capan-1 is derived from liver metastasis of pancreatic cancer. Pancreatic cancer is a hypoxic cancer and largely tropic for organs that receive blood supply (e.g., liver and lung). Upregulated oxidation-regulation associated genes with compartment switching can enhance resistance to oxidative stress of pancreatic cancer cells in liver. Besides, contact domain or loop reprogramming upregulated genes associated with PDGFR pathway or angiogenesis, which enhanced angiogenic capacity of pancreatic cancer cells, enabling them to adapt to the hepatic microenvironment surrounded by liver sinuses. Taken together, we have demonstrated that multiscale 3D epigenome reprogramming can promote pancreatic cancer metastasis by activating KRAS-associated pathways, and enhancing adaptive capability to hepatic microenvironment of pancreatic cancer cells.

However, most Hi-C maps of solid tumor are based on cell line currently [37], because cancer cells obtained from clinical specimen cannot meet the cell number requirements of Hi-C and ChIP-seq simultaneously. Compared with other solid tumor, pancreatic cancer cells consist of approximately 10–30% of the cellular components in pancreatic cancer tissue [38], which further increases the difficulty of sequencing library construction of Hi-C and ChIP-seq. Furthermore, according to NCCN guideline (www.nccn.org/patients), chemotherapy is the first-line therapy for pancreatic cancer patients with liver metastasis, because they cannot benefit from surgery. As a result, it is almost impossible to obtain comprehensive multi-omics data in the same pancreatic cancer patient with liver metastasis and we can only obtain multi-omics data from cell line currently. To avoid bias arising from cell line, we downloaded microarray/RNA-seq data of pancreatic cancer tissues from GEO. By combining our Hi-C data, we identified the key gene, LIPC, looped to Capan-1 specific enhancers and significant upregulated in metastasis lesions of pancreatic cancer. GEO datasets and our IHC analysis demonstrated that LIPC expression was significantly higher in liver metastasis compared with primary pancreatic cancer and normal pancreatic tissues. In vitro studies showed that LIPC can promote pancreatic cancer migration, invasion, and EMT process. In vivo studies further confirmed that LIPC is critical for pancreatic cancer growth and metastasis. These results emphasized the important role of LIPC in pancreatic cancer metastasis, and demonstrated that 3D epigenome reprogramming can upregulate metastasis-promoting genes to promote pancreatic cancer metastasis. Interestingly, LIPC encodes hepatic triglyceride lipase, which expresses almost exclusively in liver for fatty acid metabolism. According to opinions of Schild et al. [39], organ-specific metastatic colonization needs unique metabolic adaptations. From the perspective of metabolism, our results confirmed the speculation that 3D epigenome reprogramming in liver metastasis enables pancreatic cancer cells to adapt to the hepatic microenvironment via metabolic adaptations. The interaction among 3D epigenome, metabolism, and primary/metastatic microenvironment would be an interesting and important field for future cancer research.

## Conclusions

In summary, we reported the most comprehensive study on 3D epigenome in pancreatic cancer metastasis and identified candidate genes that promote pancreatic cancer metastasis. This forms an important resource for the pancreatic cancer research community and provides new insights into pancreatic cancer pathobiology. We anticipate that many of candidate genes may become therapeutic targets for pancreatic cancer.

## Supplementary Information

**Additional file 1**. Quality control of the next generation sequencing data in this study.

**Additional file 2**. A/B compartments identified by in situ Hi-C. (A) PC1 value of chromosome regions in three cell lines. (B) Concordance genes related to Fig. 2. (C) GO enrichment analysis of the concordance genes.

**Additional file 3**. Contact domain boundaries identified by in situ Hi-C. (A) Contact domain boundaries and their overlap in three cell lines. (B) Genes located in ± 5 kb of cell-type-specific contact domain boundaries. (C) GO enrichment analysis of the genes located in ± 5 kb of cell-type-specific contact domain boundaries.

**Additional file 4**. Contact domains identified by in situ Hi-C. (A) Contact domains in three cell lines. (B) Histone mark-enriched contact domains and FPKM of genes located in these contact domains. (C) Common and specific contact domains in Capan-1 versus PANC-1. (D) Common and specific contact domains in PANC-1 and HPNE. (E) Genes located in contact domains switched from inactive type to active type. (F) GO enrichment analysis of genes in (E).

**Additional file 5**. Loops identified by in situ Hi-C. (A) Loops in three cell lines. (B) Genes looped to Capan-1 specific enhancers, compared with PANC-1. (C) GO enrichment analysis of genes looped to Capan-1 specific enhancers. (D) Genes looped to PANC-1 specific enhancers, compared with HPNE.

**Additional file 6**. TFs involved in enhancer-promoter loops. (A) TFs enriched in Capan-1 specific enhancer NDRs. (B) TFs enriched in promoter NDRs looped Capan-1 specific enhancers. (C) TFs enriched in PANC-1 specific enhancer NDRs, compared with Capan-1. (D) TFs enriched in promoter NDRs looped PANC-1 specific enhancers, compared with Capan-1.

**Additional file 7**. Supplementary figures and tables.

## Data Availability

The raw sequence data generated in this study have been submitted to the NCBI BioProject database (https://www.ncbi.nlm.nih.gov/bioproject/) under accession number PRJNA62607. (Reviewer link: https://dataview.ncbi.nlm.nih.gov/object/PRJNA626067?reviewer=k4t73rr7jpjcnchslljs0ralt7). The processed sequence data generated in this study have been submitted to the NCBI Gene Expression Omnibus (GEO; http://www.ncbi.nlm.nih.gov/geo/) under accession number GSE149103 (Secure token: qlknsgewbdyfzsp).

## Abbreviations

Hi-C: High throughput variant of chromosome conformation capture
ATAC-seq: Assay for transposase-accessible chromatin using sequencing
ChIP-seq: Chromatin immunoprecipitation sequencing
RNA-seq: RNA sequencing
CTCF: CCCTC-binding factor
CTCFL: CCCTC-binding factor like
TSS: Transcriptional start site
BCAT1: Branched chain amino acid transaminase 1
TGFA: Transforming growth factor A
EGFR: Epidermal growth factor receptor
LIPC: Lipase C
GCKR: Glucokinase regulator
RXRA: Retinoid X receptor alpha
TF: Transcription factor
SP1: Transcription factor SP1
EGR1: Early growth response 1
KLF5: Kruppel like factor 5
E2F1: E2F transcription factor 1
MAZ: MYC associated zinc finger protein
ZFX: Zinc finger protein X-linked
