# Supplementary material for: High-resolution Hi-C maps highlight multiscale 3D epigenome reprogramming during pancreatic cancer metastasis
Source: J Hematol Oncol. 2021 Aug 4;14:120. doi: 10.1186/s13045-021-01131-0 (PMC8336101; doi:10.1186/s13045-021-01131-0)
Supplement: Supplementary file 7 — Additional file 7. Supplementary figures and tables. [file 13045_2021_1131_MOESM7_ESM.pdf]

Supplementary Materials

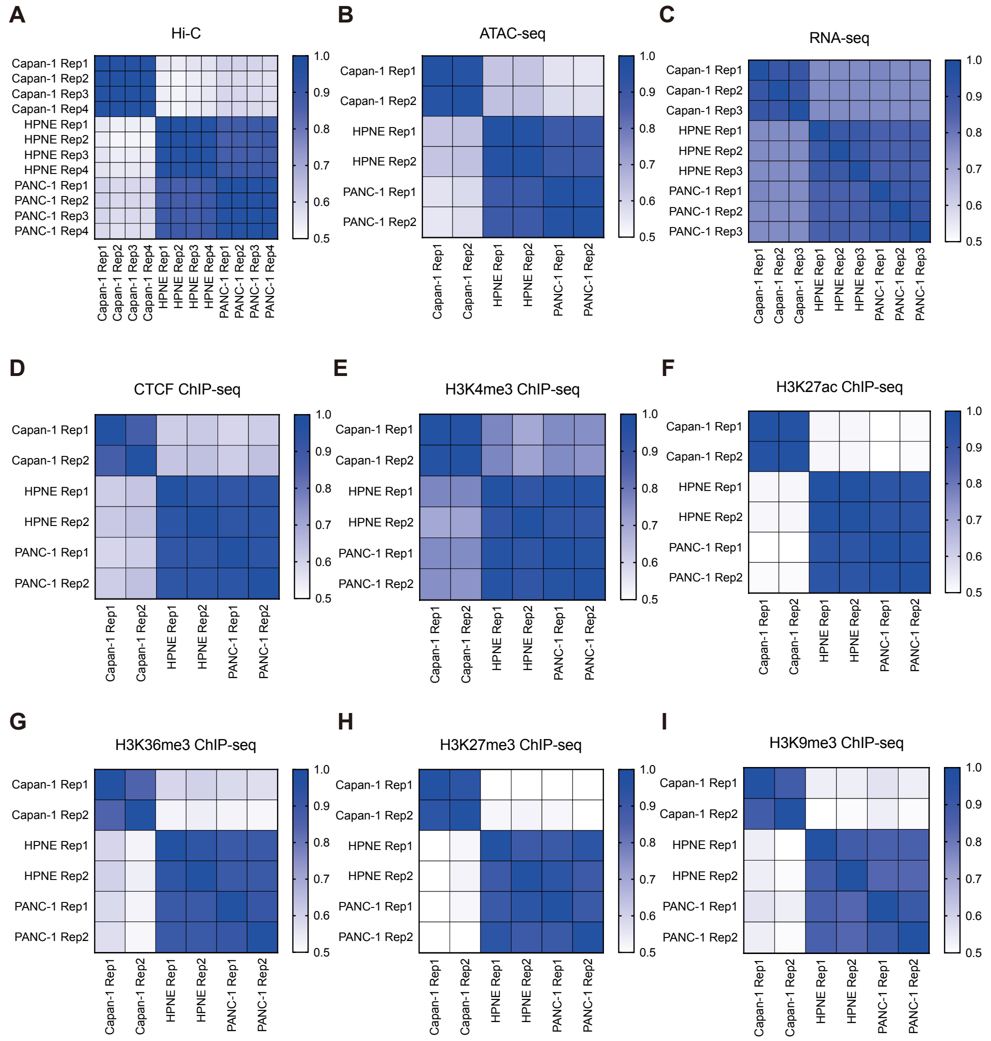

Figure S1 Multi-omics comparative analysis of normal, primary and metastatic pancreatic cancer cells. (A-I) Correlation analysis of Hi-C, ATAC-seq, RNA-seq and ChIP-seq sequencing libraries of 3 cell lines. HPNE: normal pancreatic epithelial cells; PANC-1: pancreatic cancer cells derived from primary tumor; Capan-1: pancreatic cancer cells derived from liver metastasis.

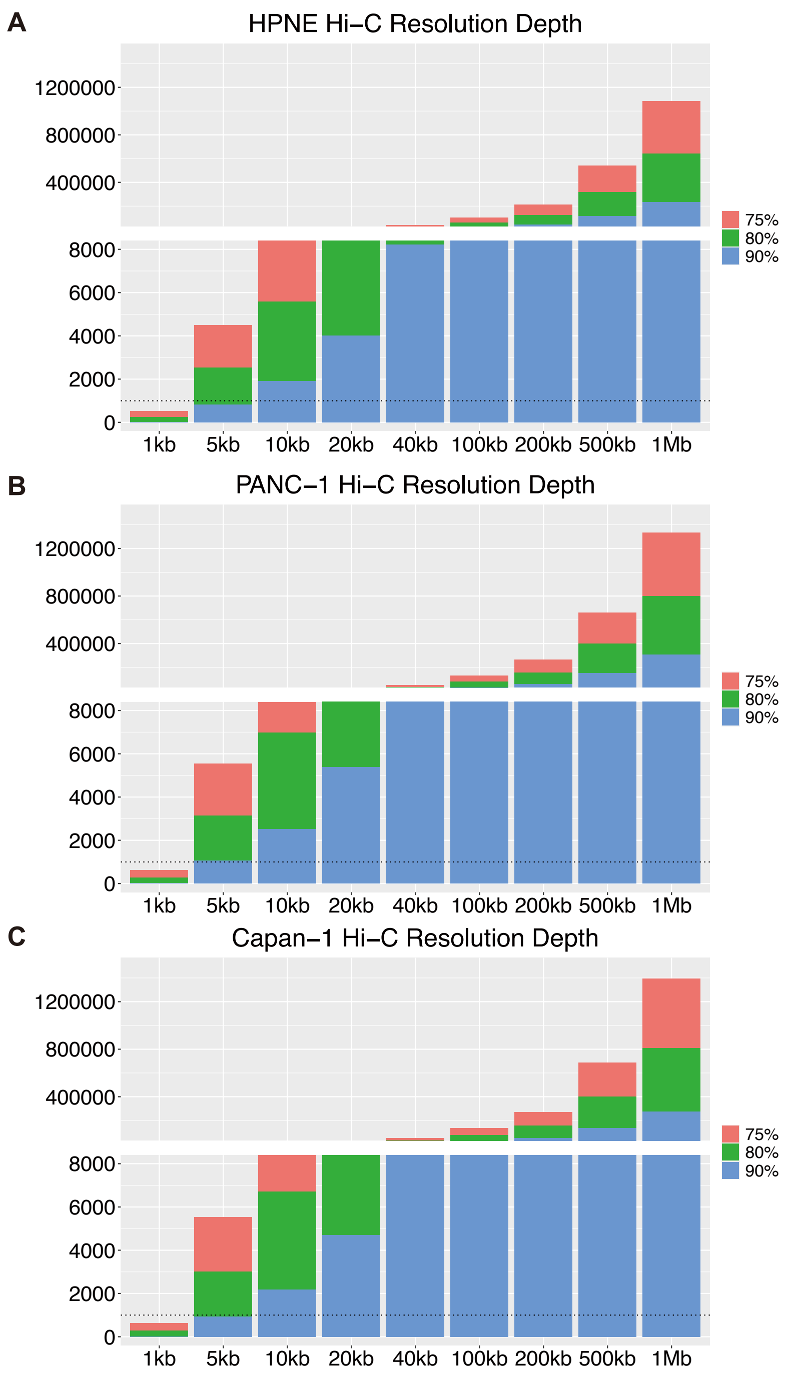

Figure S2 Hi-C resolution depth of 3 cell lines. (A) HPNE (B) PANC-1 (C) Capan-1.

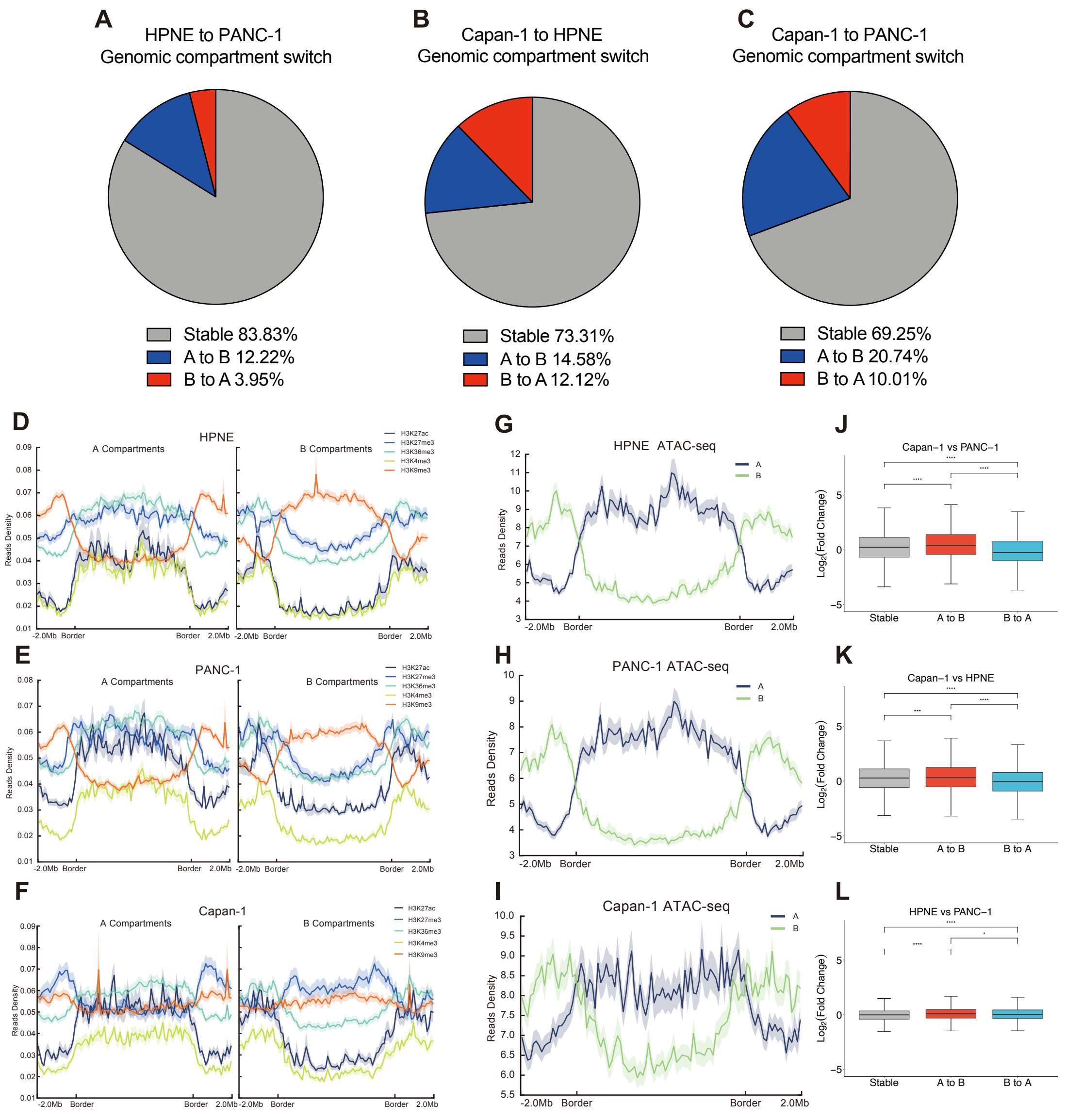

Figure S3 Association between compartment reorganization, histone modifications, chromosome accessibility, and gene expression. (A) Pie chart showing the genomic compartment changes between HPNE and PANC-1 genomes. (B) Pie chart showing the genomic compartment changes between HPNE and Capan-1 genomes. (C) Pie chart showing the genomic compartment changes between PANC-1 and Capan-1 genomes. (D-F) Profiles of active and inactive histone modifications in A and B compartments of 3 cell lines. (G-I) Chromosome accessibility profile in A and B compartments of 3 cell lines. (J-L) Relationship of compartmentalization to gene expression (Log_2(fold-change)) in 3 cell lines (Wilcoxon rank sum test, *$p<0.05$,

***p<0.001, ****p<0.0001). Whiskers of box plots are from Q1-1.5*IQR to Q3+1.5*IQR.

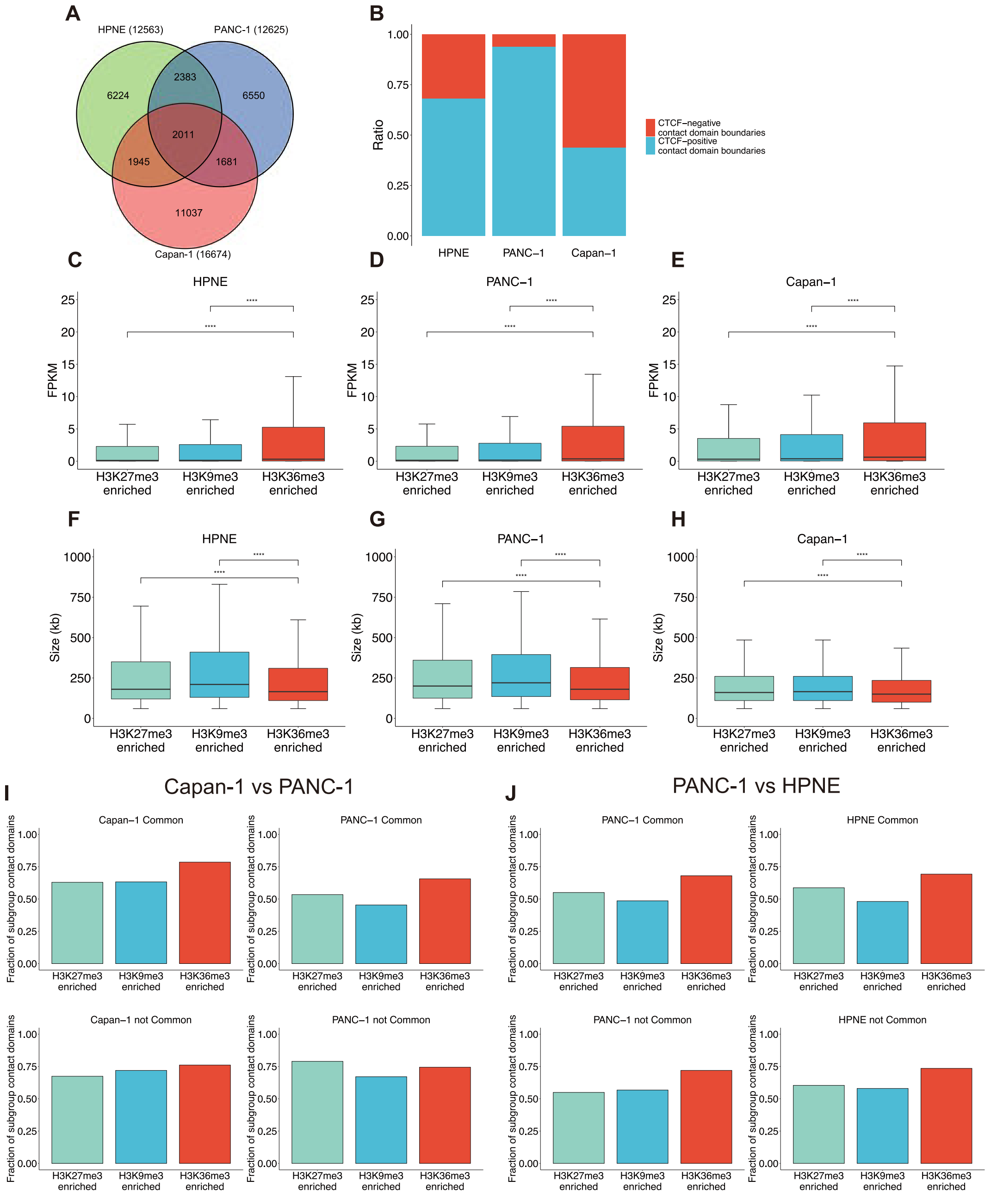

Figure S4 Alterations of contact domains and contact domain boundaries during pancreatic cancer metastasis. (A) Venn diagram showing contact domain boundaries of 3 cell lines and the overlap among them. (B) The ratio of CTCF-positive/negative contact domain boundaries in 3 cell lines. (C-E) Average expression levels of genes located in each histone mark-enriched contact domains and contact domains without

histone modifications (other) across 3 cell lines (Wilcoxon rank sum test, **p<0.01, ****p<0.0001). (F-H) Size of histone mark-enriched contact domains and other contact domains in 3 cell lines (Wilcoxon rank sum test, ****p<0.0001). Whiskers of box plots are from Q1-1.5*IQR to Q3+1.5*IQR. (I) Upper: Fraction of histone mark-enriched TAD subgroups in common TADs between primary and metastasis. Lower: Fraction of histone mark-enriched TAD subgroups in specific TADs between primary and metastasis. (J) Upper: Fraction of histone mark-enriched TAD subgroups in common TADs between normal and primary. Lower: Fraction of histone mark-enriched TAD subgroups in specific TADs between normal and primary.

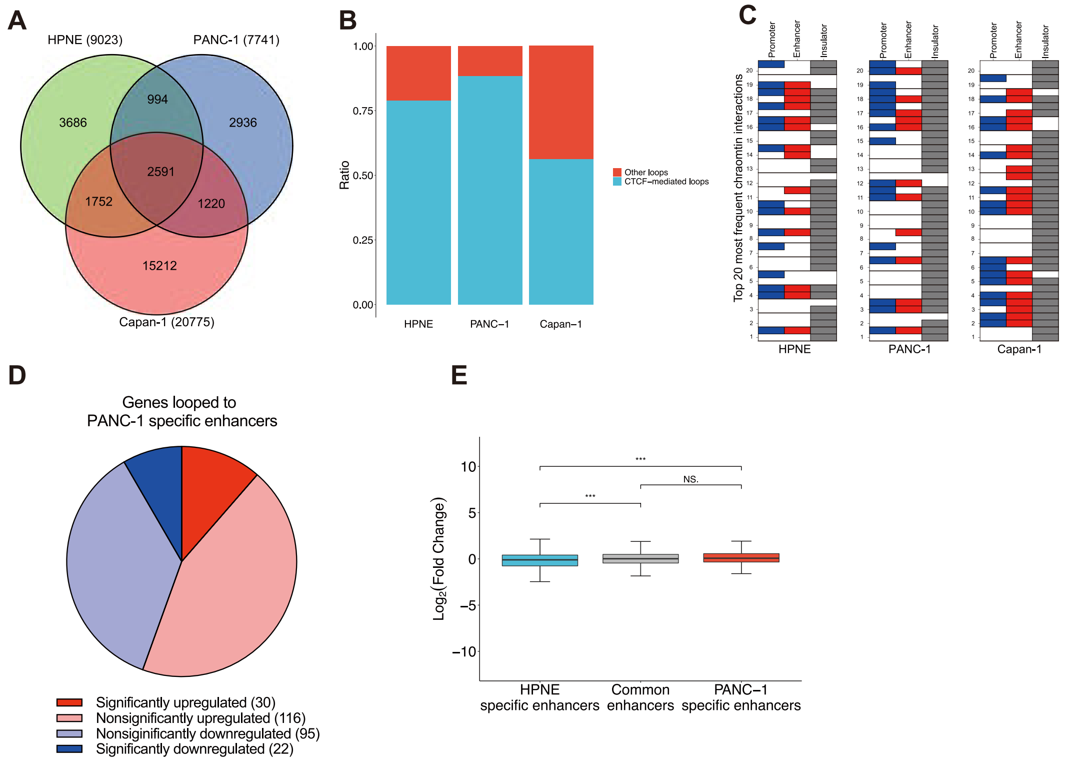

Figure S5 Loop reprogramming during pancreatic cancer metastasis. (A) Venn diagram showing TAD loops of 3 cell lines and the overlap among them. (B) The ratio of CTCF-mediated loops in 3 cell lines. (C) Top 20 most frequent loop categories for HPNE, PANC-1 and Capan-1. For each loop, the regulatory element overlapped with anchor 1 is shown on the bottom half of the rectangle and the

regulatory element overlapped with anchor 2 is shown on the top half of the rectangle. (D) Pie chart showing that genes looped to primary-specific enhancers enrich more upregulated genes. (E) Log_2(fold change) between Capan-1 and PANC-1 cells of genes that looped to primary-specific, common, and metastasis-specific enhancers (Wilcoxon rank sum test, ns $p>0.05$, ***$p<0.001$, ****$p<0.0001$).

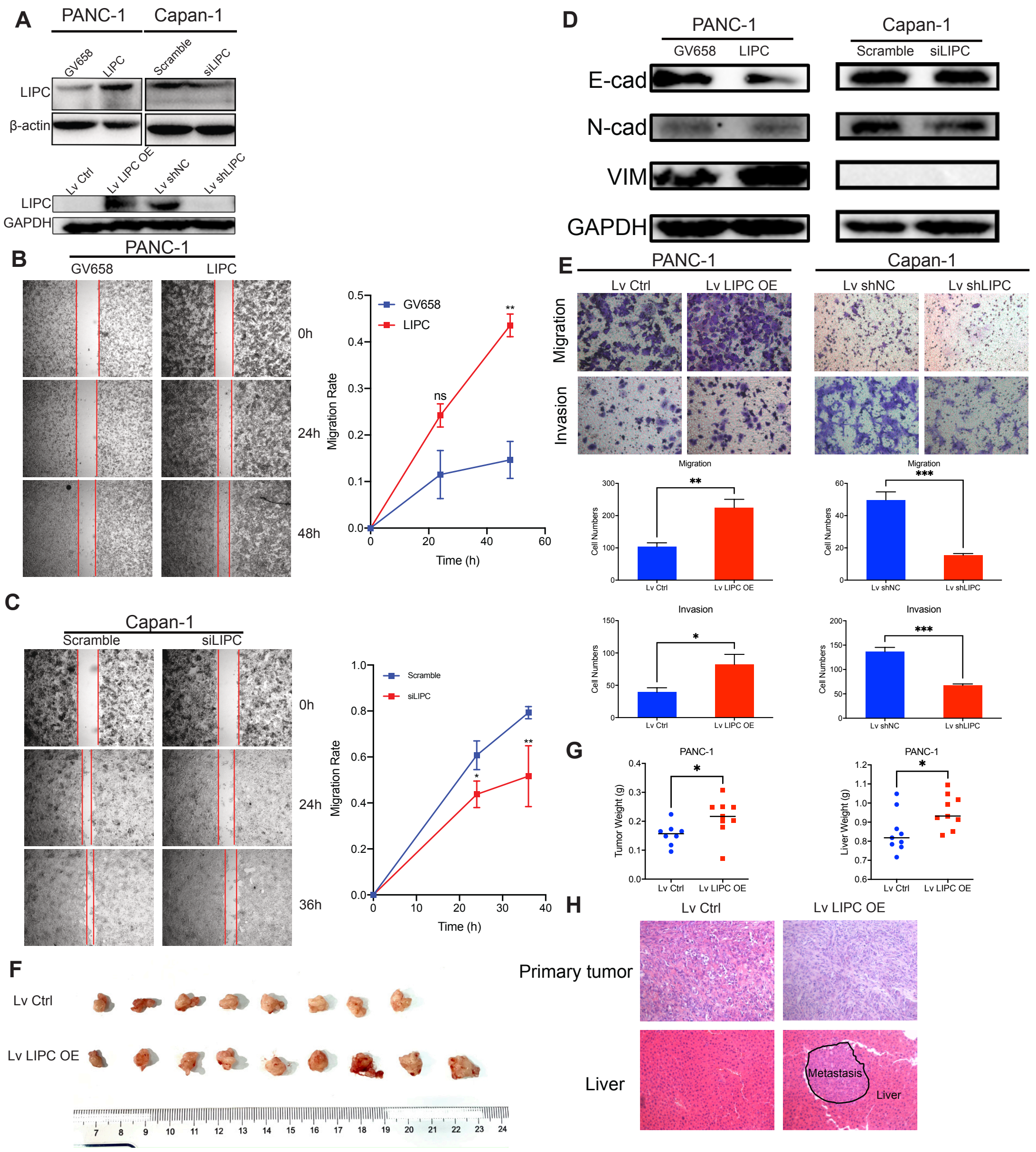

Figure S6 LIPC promotes migration and EMT process of pancreatic cancer cells. (A) Western blot of LIPC in PANC-1 with LIPC overexpression and Capan-1 with LIPC knockdown. (B-C) Wound-healing assays in Capan-1 with LIPC knockdown and

PANC-1 with LIPC overexpression. Wounds were photographed, and the migration rate (n=3) was measured by ImageJ software (Whisker: mean ± SEM, two-way ANOVA, *adj.p<0.05, **adj.p<0.01). (D) Western blot of EMT markers in PANC-1 with LIPC overexpression and Capan-1 with LIPC knockdown. (E) Transwell migration and invasion assay of PANC-1 with LIPC stably overexpression and Capan-1 with LIPC stably suppression. Representative images are shown. Maginification, ×200. Cell number of migration/invasion are shown in the bottom panel (mean + SD) (Unpaired t test, migration: p = 0.0018, invasion: p = 0.0121(PANC-1); migration: p = 0.0003, invasion: p = 0.0002). (F) Photographs of dissected tumors from orthotopic xenograft mice injected with Lv Ctrl and Lv LIPC OE (overexpression) PANC-1 cells. (G) Analysis of primary tumor weights(Left, Wilcoxon rank sum test, p = 0.0206) and liver weights (Right, Wilcoxon rank sum test, 0.0244) were calculated at the end of the experiment. (H) Representative HE staining pictures of the primary tumor and liver. (Magnification, ×200).

Table S1 Clinicopathological features of patients with primary pancreatic cancer.

Clinicopathological features	
Number of cases	87
LIPC IHC Score (mean (SD))	83.56 (27.91)
Gender (%)	
Female	33 (37.9)
Male	54 (62.1)
Age (mean (SD))	60.83 (9.36)
Smoking history (%)	34 (39.1)
Drinking history (%)	19 (21.8)
Diabetes history (%)	13 (14.9)
CA199 (mean (SD))	937.05 (2219.53)

Size (mean (SD))	3.43 (1.53)
Site (%)	
Head/Neck	48 (55.8)
Body/Tail	39 (44.2)
Differentiation (%)	
Poor	29 (37.1)
Moderate	22 (28.2)
Well	27 (34.6)
Capsule invasion (%)	80 (96.4)
Vascular invasion (%)	4 (6.5)
Bile duct invasion (%)	28 (45.9)
Neural invasion (%)	17 (27.4)
T classification (%)	
T1	16 (20.3)
T2	47 (59.5)
T3	16 (20.3)
N classification (%)	
N0	44 (50.6)
N1	43 (49.4)

Table S2 Clinicopathlogical features of pancreatic cancer patients with liver metastasis

Clinicopathological features	
Number of cases	27
LIPC IHC Score (mean (SD))	126.30 (48.01)
Gender (%)	
Female	14 (51.9)
Male	13 (48.1)
Age (mean (SD))	63.26 (9.15)
Smoking history	7 (25.9)
Drinking history	4 (14.8)

Diabetes histroy	4 (14.8)
CA199 (mean (SD))	1141.84 (1644.92)
Site	
Head/Neck	19 (70.4)
Body/Tail	8 (29.6)
Vascular invasion[1] (%)	10 (37.0)
Size of primary cancer[1] (mean (SD))	3.14 (1.28)

1. Vascular invasion and size of the primary cancer were measured by preoperative CT scanning

Table S3 Primers for ChIP-qPCR

Location	Forward primer	Reverse primer
LIPC promoter	CTGCAGCTAGCAGTGAAGTCT	CCCCGGTTGCAAATTAGATGC
LIPC enhancer 1	CCTTCGGTGTGAGTCTTTGC	TGCAACTTCACCAGCCTCTAT
LIPC enhancer 2	AGCAGCTGCCAAATTGGATGA	CTGTTCCCACTCCCTACCTC
LIPC enhancer 3	CACAGGAACGTGTTGCAAGG	TAGGGAGACAGTAGGAGCCG
LIPC enhancer 4	TGACATGGGCTGGGTGTTAT	TTCCCTGAGTTCTCCCACAC
LIPC enhancer 5	ACGAGTACACGCTATGGCAG	AGGGCTTGAGTGCCATATTT
LIPC enhancer 6	GACTTCGCTTCTCTGGGAGG	CTCCCCGCTCCCTATTGTC
LIPC enhancer 7	CGCGGGTTTGGACTTGAAGG	GTCACCTTGTACTGCCCTCTC

Table S4 Primers for 3C-qPCR

Location	Primer
LIPC promoter (constant)	AGTGCAGAGGCTGAGAAACC
LIPC enhancer 1	CCCAAGAGGTGAAAATTTGG
LIPC enhancer 2	CAGGGATGAAAAGGCAGAAA
LIPC enhancer 3	AAACAGAGGAAGCCCTACCC

LIPC enhancer 4	ACACTGTTCTGGGTGTGTGG
LIPC enhancer 5	GCAAAGTGATATTCAGCCACAA
LIPC enhancer 6	AGGCACACCTTGAGGTTTTT
LIPC enhancer 7	GGACTCAGAGCGAGTTACCTG
Negative control (Forward)	CGGGAGAAGCTGAGTCATGG
Negative control (Reverse)	TTTACAGCCTGGCCTTTGGG

Table S5 sgRNA for CRISPRi

Location	sgRNA
sgEnh1	ACAGCAAAGACTCACACCGA
sgEnh2	GCCGGCTTTAATGCCCGCGT
sgEnh3	TCGGTGGCCAGAAATTCTCG
sgEnh4	GGGGAATTAGCATACGGCCC
sgEnh5	AGCCATACGAGTACACGCTA
sgEnh6	AGAGTAGAAGTTCGGTCCCT
sgEnh7	TTGGGTACCGCCGGAGACGC
sgPro	GTCAGGAGCTAGTAACGCTA
